# Enhanced detection of circulating tumor cells using a MUC1 promoter-driven recombinant adenovirus

**DOI:** 10.3389/fonc.2024.1506968

**Published:** 2025-01-16

**Authors:** Cheng Wang, Huihui Gu, Jia Cai, Chuandong Zhu, Qin Zheng, Hanfeng Xu, Lixue Wang, Yuan Wan

**Affiliations:** ^1^ Department of Radiation Oncology, Nanjing Hospital Affiliated to Nanjing University of Chinese Medicine (The Second Hospital of Nanjing), Jiangsu, Nanjing, China; ^2^ Department of Oncology, Nanjing Hospital Affiliated to Nanjing University of Chinese Medicine (The Second Hospital of Nanjing), Jiangsu, Nanjing, China; ^3^ The Pq Laboratory of BiomeDx/Rx, Department of Biomedical Engineering, Binghamton University, Binghamton, NY, United States

**Keywords:** breast cancer, circulating tumor cell, DF3/MUC1, hTERT, adenovirus

## Abstract

**Introduction:**

Circulating tumor cells (CTCs) have attracted significant interest as a biomarker for cancer diagnosis. In this study, we judiciously constructed a recombinant MUC1-dependent adenovirus (rAdF35-MUC1) that can selectively replicate and overexpress copepod super green fluorescent proteins (copGFP) in MUC1-positive tumor cells to investigate its role in the detection of CTCs.

**Methods:**

We conducted a comparative study between rAdF35-MUC1 and the existing hTERT-dependent adenovirus (rAdF35-hTERT). Breast cancer cell lines and healthy human peripheral blood mononuclear cells (PBMCs) were infected with both viral constructs to evaluate infection efficiency and the incidence of false-positive cells. CTC Model Samples were employed to determine detection rates, and clinical samples from breast cancer patients were analyzed to preliminarily evaluate the efficacy of CTC detection in a clinical context.

**Results:**

In preclinical and clinical studies, rAdF35-MUC1 exhibited a significantly high detection efficiency for breast cancer cells, outperforming the existing hTERT-dependent adenovirus (rAdF35-hTERT), especially in detecting CTCs at low quantities. Moreover, rAdF35-MUC1 demonstrated reduced incidence of false positives in healthy PBMCs compared to rAdF35-hTERT.

**Conclusion:**

In brief, rAdF35-MUC1 emerges as a potent tool for the sensitive and specific identification of CTCs derived from breast cancer patients, holding clinical translation potential for advancing cancer (early) diagnosis, treatment monitoring, and prognosis.

## 1 Introduction

Circulating tumor cells (CTCs) are tumor cells that shed from solid tumors, intravasate into blood, and translocate to distant tissues via circulation (1). CTCs hold promises for advancing precision medicine. Numerous studies demonstrated that CTCs can be used for cancer diagnosis, treatment monitoring, and prognosis (2). Moreover, CTCs offer new insights into cancer physiopathology and relevant mechanistic research. To fully explore clinical and biological significance of CTCs, detection and isolation of CTCs is a prerequisite. Accordingly, various methods have been developed, which utilize physicochemical properties of CTCs, including size, density, deformability, surface charge, and tumor-associated antigens (3). However, CTC detection and isolation have encountered challenges due to their rarity and heterogeneity (4). In one milliliter of peripheral blood, there are <100 CTCs. Meanwhile, there are >1×10^9^ red blood cells and >1×10^6^ white blood cells, composing overwhelming “background noise” (5, 6). On the other hand, almost none of the existing physical- or immunoaffinity-based techniques has unequivocally shown clinical utility. For example, the commercially available ISET (Isolation by Size of Tumor Cells) technique is based on the size difference between CTCs and blood cells (7). This technique may miss small and/or “soft” CTCs. The low throughput further limits its clinical translation (8). So far, CellSearch, an immunoaffinity-based technique, is the only FDA-cleared product for detecting CTCs in patients with metastatic breast, colorectal, or prostate cancer (9–11). Yet, CellSearch frequently results in false negatives due to low or absent expression of EpCAM (12, 13). CellSearch cannot distinguish viable CTCs from apoptotic ones either, while only viable CTCs contribute to metastasis (14, 15). Overall, there is a clear clinical unmet need to develop innovative techniques for detection of viable CTCs.

Virus-based detection techniques may meet this need. Telomerase-selective green fluorescent protein (GFP)-containing recombinant adenovirus OBP-401 has been developed (16). OBP-401 utilizes active human telomerase reverse transcriptase (hTERT) in cancer cells to replicate and express GFP. The produced cytosolic GFP thus can “lighten” CTCs, allowing CTC detection and quantification. To further enhance detection specificity, a hTERT-dependent chimeric adenovirus Ad5F35 with CD46 recognition capability has been constructed. In addition, Ad5F35 contains a complementary sequence against miR-142-3p at the 3’-untranslated region (3’-UTR) of the target gene. The antagomirs can effectively suppress GFP expression within blood cells in a post-transcriptional manner because many leukocytes overexpress miR-142 (17). In brief, the Ad5F35 significantly enhanced infection efficiency and detection specificity, while effectively reducing false positives. Although adenovirus-based CTC detection is promising, it is noteworthy that hTERT is not consistently overexpressed in advanced tumors (18, 19). Telomerase activity of leukocytes can transiently elevate during episodes of inflammation or acute psychological stress (20). hTERT is also active in stem cells (21). These scenarios may result in false negatives or false positives.

We hypothesized that a cancer-specific promoter as a substitute for hTERT may address or reduce false findings. Previous studies have demonstrated that glycosylated protein MUC1 is overexpressed in breast, pancreatic, lung, and bladder cancers, underscoring its significance as a protein marker for cancer diagnosis and prognosis (22, 23). MUC1 promoter has critical cis-regulatory elements predominantly localized at the 5’ end (24). These elements exhibit significant tissue and cell specificity. Moreover, this region encompasses multiple transcription factor binding sites, including Sp1, AP1-4, NF-κB, E-box, and GC box, governing the downstream gene expression (25, 26). Furthermore, the transcriptional regulatory sequence of MUC1 facilitates the selective expression of target genes in MUC1-positive tumor cells (27).

The overexpression of MUC1 across various cancers makes it an ideal target for selective immunotherapies. Adenovirus-based constructs regulated by the MUC1 promoter have demonstrated significant promise, particularly in immunotherapy and vaccine development. For example, oncolytic adenoviruses targeting both the MUC1 and hTERT promoters have shown enhanced tumor targeting and the ability to overcome tumor heterogeneity in preclinical models (28). Furthermore, MUC1-driven adenoviruses expressing the sodium iodide symporter (NIS) exhibit substantial therapeutic potential, particularly in ovarian cancer (29). Clinical trials, such as a Phase I study of a recombinant adenovirus vaccine for metastatic prostate cancer, have reported both favorable tolerance and promising efficacy (30). These findings underscore the significant potential of MUC1-based strategies, not only for cancer treatment but also for improving the sensitivity and specificity of CTC detection.

Taken together, we developed a MUC1-dependent adenovirus (rAdF35-MUC1), which includes the fiber protein for CD46 binding and a complementary sequence for producing antagomirs against miR-142-3p. This combination could achieve unprecedented CTC detection sensitivity and specificity. In parallel, hTERT-dependent adenovirus (rAdF35-hTERT) as a negative control was constructed. Subsquently, we evaluated their performances in CTC detection for breast cancer using both CTC model and clinical samples.

## 2 Materials and methods

### 2.1 Cell culture

The human breast cancer cell lines MDA-MB-231, MDA-MB-468, and MCF-7, as well as the human normal breast epithelial cell line MCF-10A, were procured from (Procell, China). The MDA-MB-231, MDA-MB-468, and MCF-7 cell lines were cultured in DMEM/F12 medium supplemented with 10% fetal bovine serum (FBS). The MCF-10A cell line was cultured in DMEM/F12 medium supplemented with 5% horse serum (HS), 10 µg/ml insulin, 20 ng/ml epidermal growth factor (EGF), and 0.5 µg/ml hydrocortisone. All cell lines were maintained in a cell culture incubator at 37°C with 5% CO_2_.

### 2.2 Design and construction of rAdF35-MUC1 and rAdF35-hTERT

**Figure 1:**
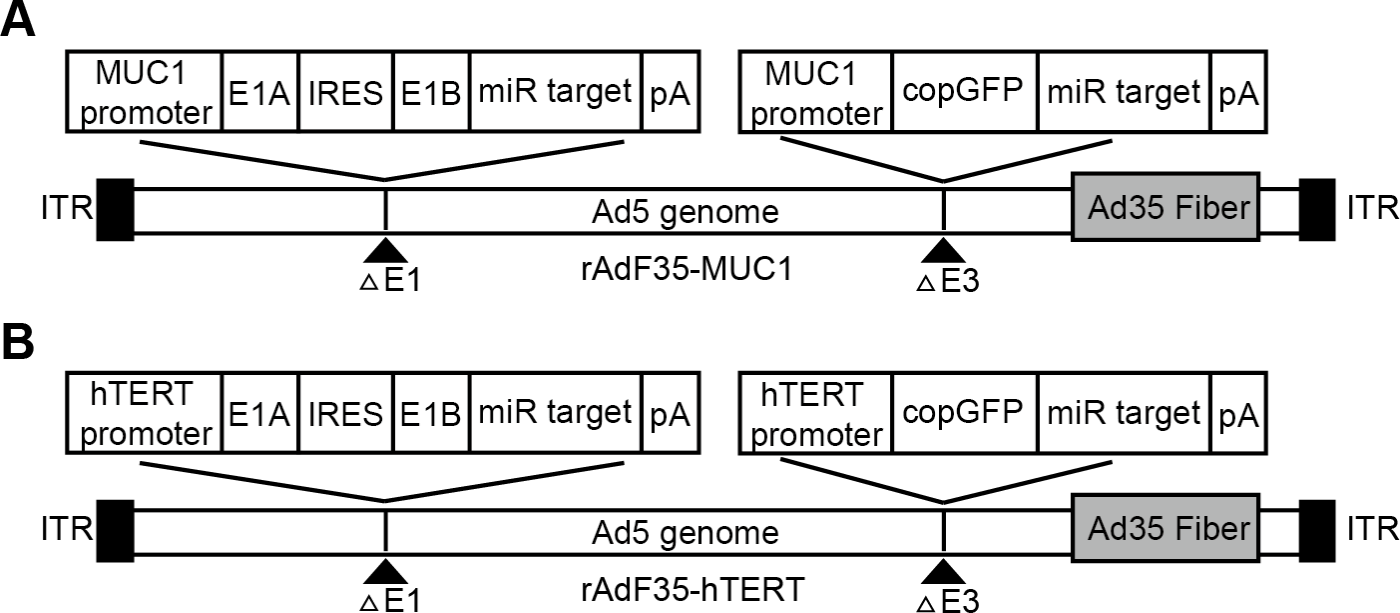
The schematic representation of the two recombinant adenoviruses. **(A)** rAdF35-MUC1 and **(B)** rAdF35-hTERT (ITR, internal terminal repeat; pA, SV40 poly(A) signal).

A recombinant shuttle plasmid, designated as pMUC1-E1A-IRES-E1B-miR, was constructed using the pDC316 adenoviral shuttle plasmid (MiaoLingBio, China) as a template. This plasmid utilizes the MUC1 promoter to facilitate the expression of the E1A and E1B genes, which are linked via an IRES. Furthermore, four oligonucleotides encoding sequences complementary to miR-142-3p were inserted into the 3’-UTR of the E1B gene. To construct the plasmid pMUC1-copGFP-miR, the E1-IRES-E1B genes in pMUC1-E1A-IRES-E1B-miR were replaced with the cDNA of copGFP. Furthermore, the adenoviral helper plasmid pBHGlox(Δ)E1,3Cre (MiaoLingBio, China) underwent modification through the replacement of the fiber gene from adenovirus serotype 5 with that from serotype 35, producing the chimeric adenoviral helper plasmid pBHG-Ad5F35. Subsequently, an expression cassette driven by the MUC1 promoter, containing the copGFP gene and miR-142-3p complementary sequences, was inserted into the E3 region, resulting in the construction of pBHG-Ad5F35-MUC1-copGFP-miR (**Figure 1A**). The adenoviral helper plasmid (pBHG-Ad5F35-MUC1-copGFP-miR) and the recombinant shuttle plasmid (pMUC1-E1A-IRES-E1B-miR) were co-transfected into HEK293 cells (Puno Bio, China) utilizing the jetPRIME transfection reagent (Polyplus, France). In the control group (**Figure 1B**), rAdF35-hTERT was constructed following the same protocol. The recombinant adenoviruses were subsequently amplified in HEK293 cells and purified via cesium chloride gradient centrifugation. Viral titers were quantified using the TCID50 method.

### 2.3 Design of rAdF35-MUC1

Our rAdF35-MUC1 contains the Ad35 fiber protein which can specifically bind to CD46 on cell outer membranes, allowing rAdF35-MUC1 to infect various tissue-specific cancer cells. The E1A and E1B genes, connected via an internal ribosome entry site (IRES), are regulated by the MUC1 promoter and integrated into the E1 region of a replication-deficient adenovirus. Concurrently, the copepod super green fluorescent protein (copGFP) gene, also under the regulation of the MUC1 promoter, is inserted into the E3 region. Furthermore, four oligonucleotides encoding sequences complementary to miR-142-3p are incorporated into the 3’-untranslated region (3’-UTR) of both the E1B and copGFP genes to selectively suppress their expression in blood cells. The control virus rAdF35-hTERT possesses an identical structural configuration to rAdF35-MUC1, with the sole distinction being the utilization of the hTERT promoter (**Figure 1**).

### 2.4 Real-time RT-PCR

Total RNA was isolated from cultured cells using TRIZOL (Invitrogen, USA). Subsequently, cDNA libraries were synthesized through reverse transcription using the PrimeScript™ RT Reagent Kit with gDNA Eraser (TAKARA, Japan). qRT-PCR was conducted employing the TB Green^®^ Premix Ex Taq™ II (Tli RNaseH Plus) (TAKARA, Japan) on the BIORAD CFX Connect RT system. The amplification protocol was executed as follows: an initial denaturation step at 95°C for 30 seconds, succeeded by 40 cycles of amplification comprising denaturation at 95°C for 5 seconds and annealing/extension at 60°C for 34 seconds. PCR amplification utilized the following specific primers: MUC1 F: 5′-GTG CCA TTC CAC TCC ACT CA-3′ and R: 5′-CTG AAC TCC CAG CTC ACC AG-3′; GAPDH F: 5′-GTC TTC ACC ACC ATG GAG AA-3′ and R: 5′-TAA GCA GTT GGT GGT GCA G-3′. Each sample was analyzed in triplicate, with GAPDH employed as the reference gene for normalization of PCR data. The normalized expression ratio for each sample was calculated by dividing its value by that of the MCF-10A cell line. Gene expression was determined using the -2^△△^Ct method, with Ct values greater than 35 considered negative.

### 2.5 Analysis of copGFP expression in human cancer cell lines

A total of 1.8×10^5^ cancer cells were seeded into each well of a 24-well plate and subsequently infected with recombinant adenovirus at a multiplicity of infection (MOI) of 10 per well. Following a 24-hour incubation period at 37°C, the cancer cells were harvested and subjected to washing. The expression of copGFP was analyzed using the Attune^®^ NxT flow cytometer (ThermoFisher, USA). The MOI of 10 was selected based on preliminary experiments (**Supplementary Figure S1**), which demonstrated that at this dose, the cells maintained high viability while achieving optimal infection efficiency. Cells were imaged using a Leica DMI8 microscope (Leica, Germany).

### 2.6 Analysis of copGFP expression in human leukocytes

Four milliliters of peripheral blood were collected from healthy volunteers, and PBMCs were subsequently isolated via Ficoll density gradient centrifugation. Subsequently, recombinant adenovirus was introduced to approximately 1×10^5^ PBMCs at a multiplicity of infection (MOI) of 10. The cells were incubated at 37°C for 24 hours, after which they were stained with anti-CD45 antibody (eBioscience, USA) and Hoechst 33342 (Beyotime, China). After fixation with 4% paraformaldehyde (Beyotime, China), images were acquired using the ImageXpress Micro XL high-content imaging system (Molecular Devices, USA). Captured images were analyzed with MetaXpress software (Molecular Devices, USA) to determine the total number of cells by nuclear count and to quantify the number of single, double or triple fluorescent positive cells among the nuclear stained cells. copGFP+/CD45+ cells were identified as false positives.

### 2.7 Detection of cancer cells spiked into human PBMCs

Aliquots containing 10, 50, 100, and 200 MCF-7 cells were introduced into the peripheral blood of healthy volunteers to mimic CTCs. PBMCs were isolated using Ficoll density gradient centrifugation, then incubated with recombinant adenovirus, stained, and imaged following the aforementioned protocols. Tumor cells were identified as copGFP+/CD45- cells.

### 2.8 Detection of CTCs in blood derived from breast cancer patients

Four milliliter of blood samples were obtained from breast cancer patients utilizing EDTA-K2 anticoagulant tubes. PBMCs were isolated, incubated with recombinant adenovirus, stained, and imaged following the aforementioned protocols. CTCs and false-positive cells were characterized as copGFP+/CD45- and copGFP+/CD45+ cells, respectively. This study received approval from the Ethics Review Committee of Nanjing Second Hospital (2016-LY-kt038), and written informed consent was obtained from all participating patients.

### 2.9 Statistical analysis

Statistical analyses were conducted utilizing GraphPad Prism 8. Data are expressed as mean ± SD. Differences between two groups were assessed using a t-test, whereas one-way analysis of variance (ANOVA) was applied for comparisons across multiple groups. A significance threshold of 0.05 was established, with p<0.05 deemed statistically significant.

## 3 Results

### 3.1 Expression levels of MUC1 in cultured tumor cell lines and PBMCs

**Figure 2:**
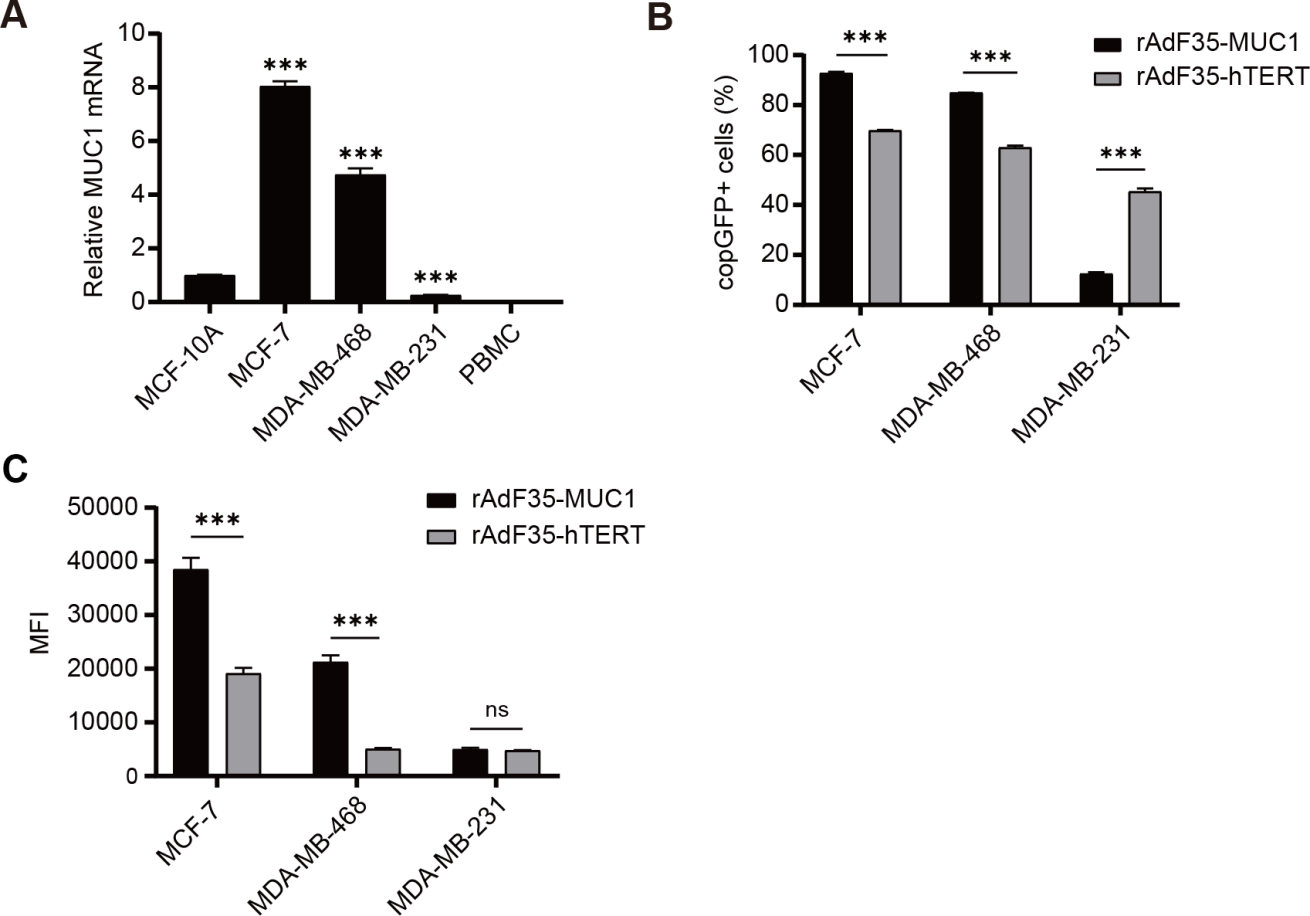
MUC1 mRNA expression and selective replication of recombinant adenoviruses in various cells. **(A)** The mRNA levels of MUC1 in various cells were quantified using RT-qPCR, and the ratios of MUC1 to GAPDH levels were determined. To facilitate comparison, The ratio in MCF-10A cells was normalized to a value of 1. Subsequently, breast cancer cells were infected with conditionally replicating adenoviruses at a multiplicity of infection (MOI) of 10. **(B)** The percentage of cells expressing copGFP in each group was determined, respectively. **(C)** The MFI of individual copGFP-positive cells were assessed by flow cytometry after a 24-hour incubation period. Data are presented as mean ± SD; n=3; ***p < 0.001; ns, not significant.

We evaluated MUC1 mRNA expression in three breast cancer cell lines, *i.e.*, MCF-7, MDA-MB-468, and MDA-MB-231, a normal human breast epithelial cell line, *i.e.*, MCF-10A, and PBMCs derived from a healthy volunteer, respectively. RT-qPCR data revealed that MUC1 mRNA expression levels in MCF-7 and MDA-MB-468 cells were ~8-fold and ~4.8-fold higher, respectively, compared to MCF-10A cells. In contrast, MUC1 mRNA expression in MDA-MB-231 cells was ~3.77-fold lower than that observed in MCF-10A cells. MUC1 mRNA was undetectable in PBMCs (**Figure 2A**).

### 3.2 Detection of breast cancer cells *in vitro*

Four cell lines and PBMCs were exposed to rAdF35-MUC1 and rAdF35-hTERT, respectively, to determine whether the two engineered adenoviruses can efficiently infect cells and express copGFP. Compared to rAdF35-hTERT, rAdF35-MUC1 “lightened” ~1.33-fold more MCF-7 cells and ~1.35-fold more MDA-MB-468 cells. Conversely, ~3.64-fold more copGFP-positive MDA-MB-231 cells were identified in rAdF35-hTERT group than those in rAdF35-MUC1 group (**Figure 2B**). Moreover, mean fluorescence intensity (MFI) of copGFP-positive MCF-7 and MDA-MB-468 cells induced by rAdF35-MUC1 was ~2-fold and ~4.22-fold higher, respectively, than that of the rAdF35-hTERT treated groups (**Figure 2C**). While rAdF35-hTERT induced ~3.64-fold more copGFP-positive MDA-MB-231 cells, the MFI between rAdF35-MUC1 and rAdF35-hTERT groups did not show significant difference (**Figure 2C**). Overall, these findings suggest that both adenoviruses can infect CD46-expression cancer cells and synthesize copGFP. The copGFP expression levels and the amount of copGFP-positive cells are influenced by promoter activity and cell-specific regulatory mechanisms.

### 3.3 rAdF35-MUC1 significantly reduces false positives

**Figure 3:**
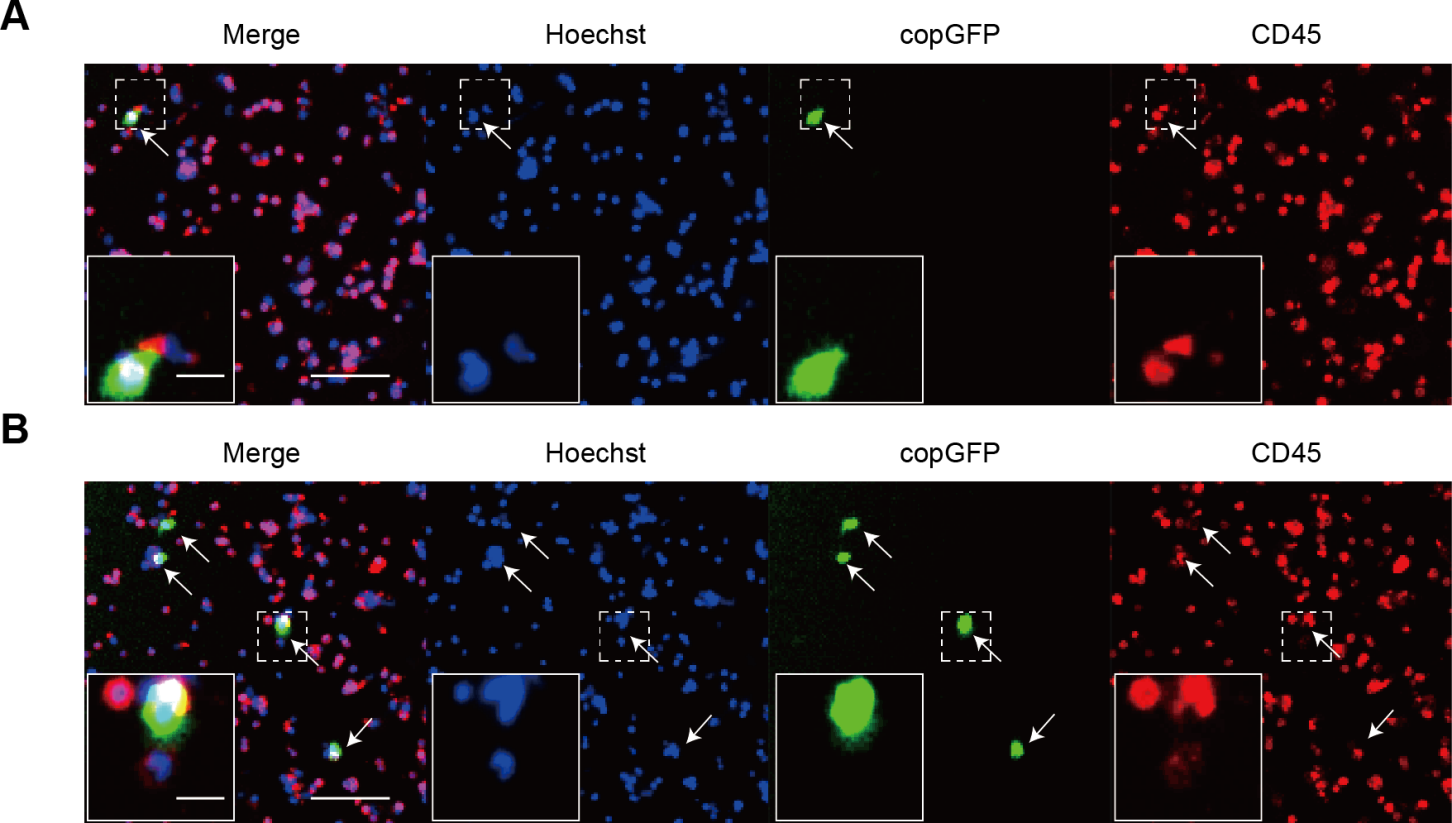
The expression of copGFP in PBMCs derived from healthy donors. **(A, B)** illustrate PBMCs infected with rAdF35-MUC1 and rAdF35-hTERT, respectively. False positives were indicated by white arrowheads. Scale bars, 100 μm and 20 μm (inserts).

PBMCs isolated from healthy donors were exposed to rAdF35-MUC1 and rAdF35-hTERT, respectively. copGFP+/CD45+ PBMCs were detected (**Figures 3A, B**). copGFP+/CD45+ PBMC proportion in rAdF35-MUC1 group was 0.0011%, whereas the proportion in rAdF35-hTERT group was 0.0036% (**Supplementary Figure S2**). The ~3.3-fold difference suggests that the MUC1 promoter robustly inhibits copGFP expression in PBMCs, thereby rAdF35-MUC1 can minimize false positives.

### 3.4 Determination of detection rates using CTC model samples

**Figure 4:**
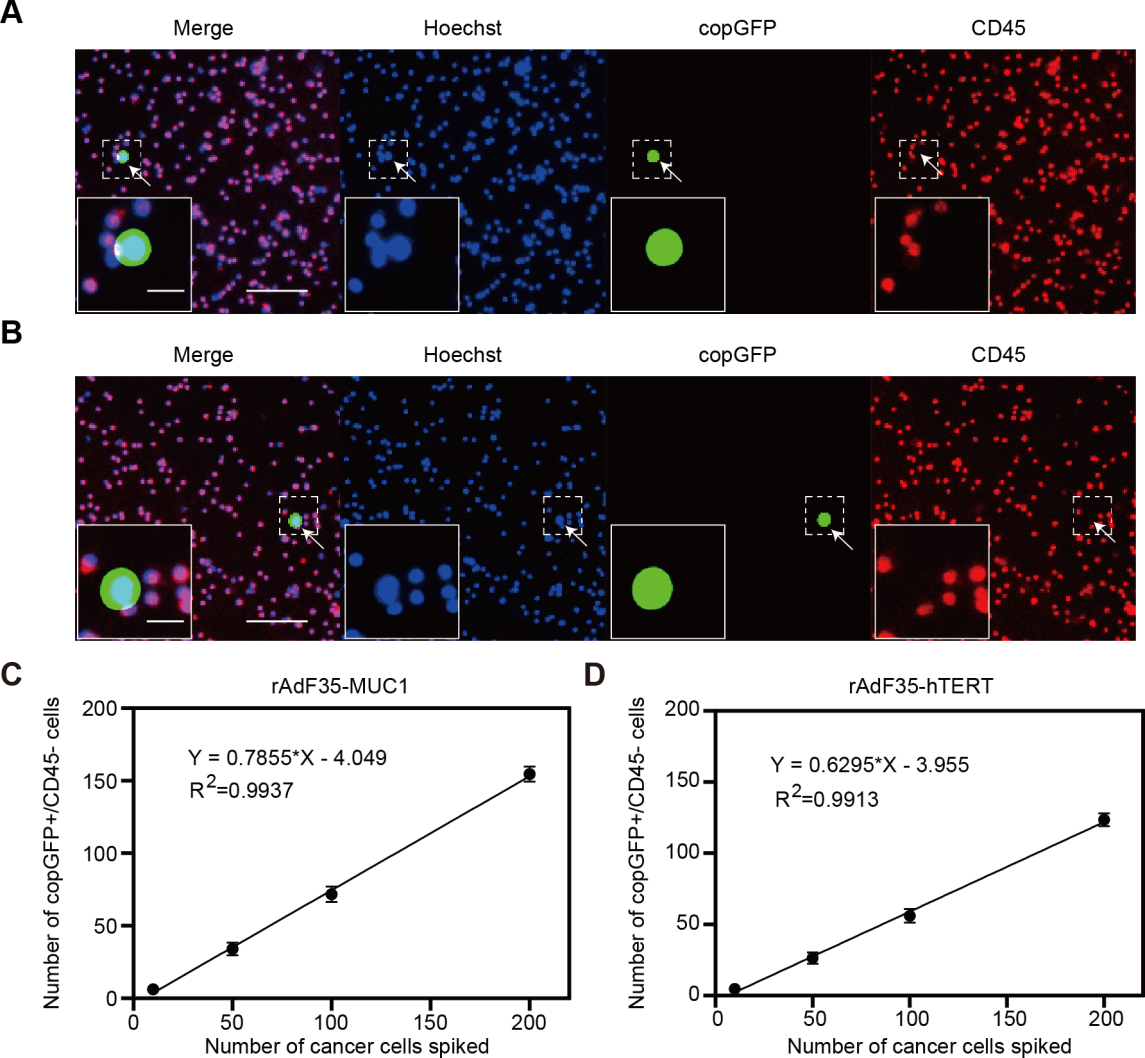
Detection of breast cancer cells in CTC model samples. MCF-7 cells were introduced into PBMCs and subsequently exposed to adenoviruses 24 hours. Merged fluorescence imaging of cells infected with **(A)** rAdF35-MUC1 and **(B)** rAdF35-hTERT was obtained. copGFP+/CD45- cells were indicated by white arrowheads. Scale bars, 100 μm and 20 μm (inserts). **(C, D)** A linear regression analysis was conducted to show the relationship between the number of spiked MCF-7 cells and the number of copGFP+/CD45- cells. Data are presented as mean ± SD (n=5).

Given that MCF-7 cells are a well-established model in breast cancer research and demonstrated high MUC1 expression in our experiments, we selected MCF-7 cells for the generation of the CTC model samples. Varying quantities of MCF-7 cells were introduced into 4 ml of peripheral blood obtained from healthy donors. These CTC model samples were treated with rAdF35-MUC1 and rAdF35-hTERT, respectively (**Figures 4A, B**). Subsequently, the presence of copGFP+/CD45- cells was calculated. The detection rate of rAdF35-MUC1 ranged from 62% to 77.3%, while the detection rate of rAdF35-hTERT ranged from 48% to 60.18% (**Tables 1**, **2**). Linear regression analysis showed a significant correlation between the number of copGFP+/CD45- cells and the number of spiked MCF-7 cells, with an R² value of 0.9937 in the rAdF35-MUC1 group (**Figure 4C**). In the rAdF35-hTERT group, a similarly significant correlation was observed (R² = 0.9913) (**Figure 4D**). These findings suggest that the number of copGFP+/CD45- cells accurately reflects the quantity of CTCs in peripheral blood, and that rAdF35-MUC1 achieves a significantly higher detection rate.

**Table 1:** Recovery rate of MCF-7 cells detected by rAdF35-MUC1 (n=5).

Number of tumor cells	10	50	100	200
Mean	6.2	34	71.8	154.6
Detection rate (%)	62%	68%	71.8%	77.3%

**Table 2:** Recovery rate of MCF-7 cells detected by rAdF35-hTERT (n=5).

Number of tumor cells	10	50	100	200
Mean	4.8	26.4	56	123.6
Detection rate (%)	48%	52.8%	56%	61.8%

### 3.5 Detection of CTCs in breast cancer patients’ blood samples

**Figure 5:**
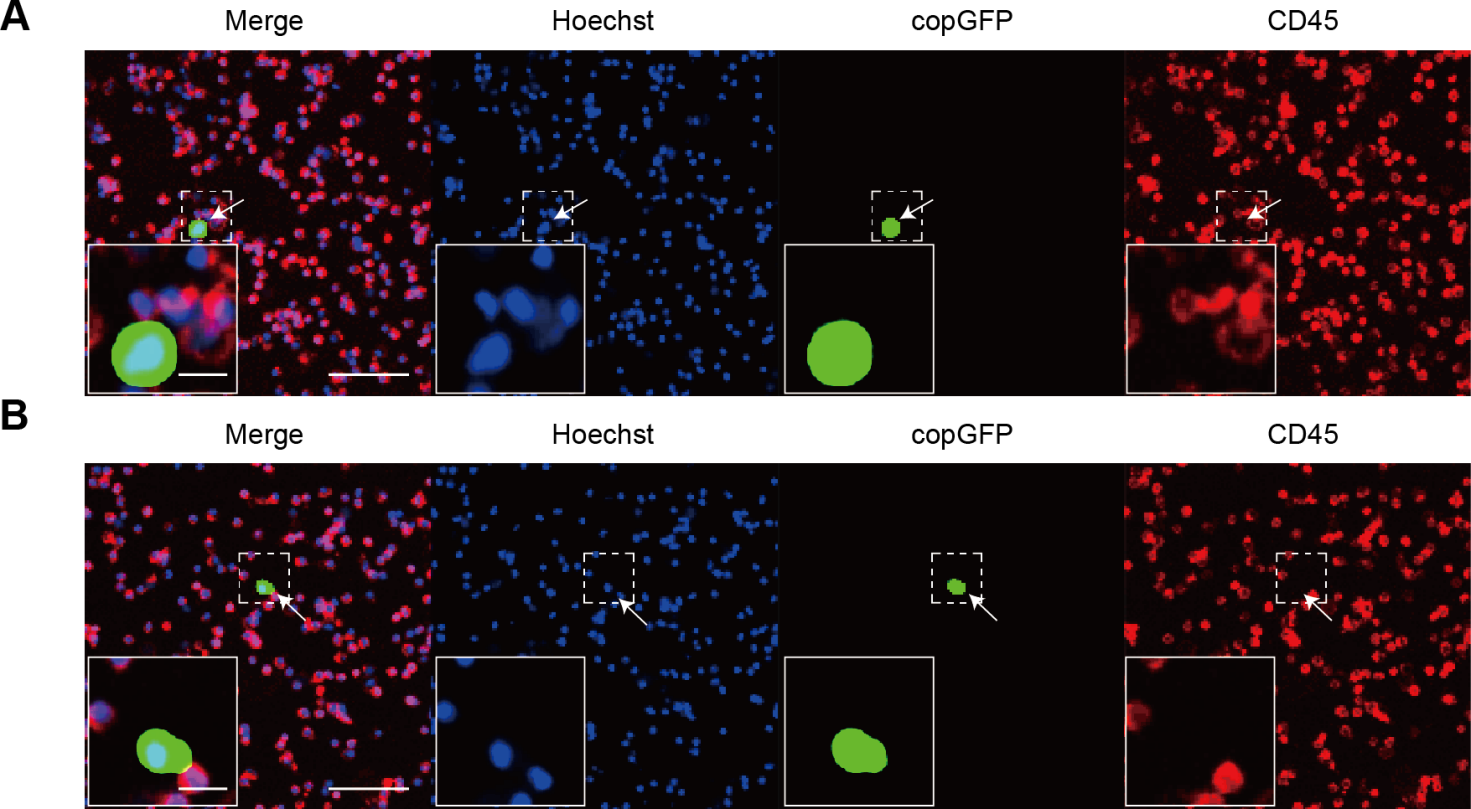
The detection of CTCs in breast cancer patients using recombinant adenoviruses. **(A, B)** Illustrate cells infected with rAdF35-MUC1 and rAdF35-hTERT, respectively, with CTCs indicated by white arrowheads. Scale bars, 100 μm and 20 μm (inserts).

Blood samples from 15 patients with breast cancer were processed by rAdF35-MUC1 and rAdF35-hTERT, respectively. Both adenoviruses successfully detected CTCs (**Figures 5A, B**, **Table 3**). In rAdF35-MUC1 group, 9 (60%) were found to be CTC-positive, with a range of 0 to 13 cells and an average of 2.93 CTCs per sample. The proportion of false positives was 27.46%, with a range of 0 to 5 cells and a mean of 1.33 false-positive cells per sample. In contrast, rAdF35-hTERT identified CTCs in 7 patients (46.7%), with a range of 0 to 11 cells and an average of 2.13 CTCs per sample. The false-positive rate for rAdF35-hTERT was determined to be 73.02%, with a range spanning from 0 to 21 cells and an average of 5.27 false-positive cells per sample. Additionally, an analysis of the percentage of CTCs among copGFP+ cells in CTC-positive samples revealed values of 80.6% for rAdF35-MUC1 and 38.5% for rAdF35-hTERT (**Supplementary Figure S3**). These findings indicate that rAdF35-MUC1 is effective in CTCs and exhibits superior detection efficiency and accuracy compared to rAdF35-hTERT.

**Table 3:** Detection of CTCs in patients with breast cancer.

Patient No.	Total copGFP+ cells	CTCs	False-positive cells	True-positive cells (%)	False-positive cells (%)
rAdF35-MUC1(n=15)						
1	5	4	1	80.00	20.00
2	13	8	5	61.54	38.46
3	2	2	0	100.00	0.00
4	0	0	0	/	/
5	0	0	0	/	/
6	4	3	1	75.00	25.00
7	6	4	2	66.67	33.33
8	10	7	3	70.00	30.00
9	0	0	0	/	/
10	3	0	3	0.00	100.00
11	0	0	0	/	/
12	18	13	5	72.22	27.78
13	1	1	0	100.00	0.00
14	0	0	0	/	/
15	2	2	0	100.00	0.00
Average	4.27	2.93	1.33	72.54	27.46
SD	5.44	3.81	1.84	29.34	29.34
rAdF35-hTERT(n=15)						
1	4	0	4	0.00	100.00
2	26	5	21	19.23	80.77
3	0	0	0	/	/
4	2	1	1	50.00	50.00
5	7	0	7	0.00	100.00
6	6	2	4	33.33	66.67
7	13	6	7	46.15	53.85
8	14	4	10	28.57	71.43
9	0	0	0	/	/
10	8	3	5	37.50	62.50
11	0	0	0	/	/
12	20	11	9	55.00	45.00
13	0	0	0	/	/
14	11	0	11	0.00	100.00
15	0	0	0	/	/
Average	7.4	2.13	5.27	26.98	73.02
SD	8.06	3.2	5.87	21.29	21.29

## 4 Discussion

The quantification of CTCs is recognized as a pivotal clinical prognostic biomarker across a spectrum of cancers (10, 31), thereby underscoring the imperative for the development of efficient and precise CTC detection methodologies in cancer diagnosis. This study aimed to evaluate the detection efficacy of rAdF35-MUC1. The results demonstrate that rAdF35-MUC1 exhibits markedly superior sensitivity and specificity for CTC detection compared to rAdF35-hTERT, particularly at low CTC concentrations. This outcome is likely attributable to the highly selective expression of the MUC1 promoter in tumor cells, thereby underscoring the potential utility of rAdF35-MUC1 in CTC detection. This is particularly significant for early cancer detection and clinical screening in patients with a minimal tumor burden.

MUC1 is a transmembrane glycoprotein that exhibits low or negligible expression in normal breast tissue but is markedly upregulated in breast cancer cells (32). This dysregulated expression is predominantly controlled at the transcriptional level (33). Moreover, a study encompassing 226 breast cancer patients identified CTCs in 31% of the cancer samples (34). Subsequent analysis of these CTC-positive samples indicated the overexpression of three biomarkers, including MUC1. Notably, 60% of the samples exhibited overexpression of at least two biomarkers, with MUC1 being significantly overexpressed in 24% of the CTC-positive samples. These findings suggest that the aberrant expression of MUC1 in CTCs is significant. Consequently, this study employs the MUC1 promoter to drive the recombinant adenovirus, aiming for more precise targeting of breast cancer cells and thereby enhancing the detection of CTCs.

In this experiment, the proportion of copGFP-positive cells and the MFI resulting from rAdF35-MUC1 infection in MDA-MB-468 and MCF-7 breast cancer cells were significantly higher than those observed with rAdF35-hTERT. These differences in fluorescence intensity, attributable to the distinct promoters, underscore the varying activity of the promoters (35). Specifically, the MUC1 promoter demonstrates superior transcriptional activity in these MUC1-overexpressing tumor cells compared to the hTERT promoter. In MDA-MB-231 cells, the hTERT promoter likely induces a higher proportion of copGFP-positive cells due to the high telomerase activity typical of triple-negative breast cancer (TNBC) cells like MDA-MB-231 (36). In contrast, within MDA-MB-231 cells, the proportion of copGFP-positive cells and the MFI following rAdF35-MUC1 infection were markedly diminished. This observation suggests that the MUC1 promoter lacks the capacity to drive high transcriptional activity in MUC1-negative or MUC1-low activity cells. These findings are in agreement with previously reported studies (37, 38). However, the similar MFI between the two groups suggests that rAdF35-hTERT drives broader but weaker expression, while rAdF35-MUC1, though affecting fewer cells, induces a more concentrated expression in those it does activate. Overall, our rAdF35-MUC1 exhibits enhanced selective replication and expression capabilities in tumor cells that overexpress MUC1.

The infection of healthy human PBMCs with rAdF35-MUC1 resulted in a significant reduction in the number of false positives, suggesting that the MUC1 promoter offers high specificity for CTC detection. This finding is particularly significant, given that telomerase activity in leukocytes may transiently elevate in response to conditions such as inflammation, infection, and acute psychological stress (20, 39). Cancer patients frequently exhibit chronic inflammatory responses (40), which could result in non-specific activation of the hTERT promoter and consequently increase the incidence of false positive cells. Therefore, using the MUC1 promoter can effectively address the limitations associated with the hTERT promoter under inflammatory or stress conditions, thereby reducing false positives. Detection results from clinical samples further substantiate the substantial efficacy of the MUC1 promoter in CTC detection.

CellSearch, the only FDA-approved method for detecting CTCs, exhibits several significant limitations. One major challenge lies in its reliance on EpCAM expression, which limits its ability to detect EpCAM-negative tumor cells, such as breast cancer cells with stem cell-like properties (41). During epithelial-mesenchymal transition (EMT), a process central to cancer metastasis, tumor cells frequently downregulate or lose EpCAM expression, further reducing the method’s effectiveness in identifying specific CTC subsets (42). Additionally, CellSearch is less effective in isolating breast cancer CTCs that lack cytokeratins 8, 18, and 19 (43). Alternative separation techniques based on the physical properties of CTCs also face challenges, as they typically assume that tumor cells are larger than normal white blood cells. However, in metastatic breast cancer and other cancers, CTCs often exhibit size ranges that overlap with or are smaller than those of white blood cells, diminishing the efficacy of size-based separation (44). Moreover, residual cell debris and fragments in blood samples can obstruct filtration pores, further compromising the performance of these separation methods (45).

In recent years, recombinant adenovirus-based technologies have demonstrated significant potential for detecting CTCs. Kojima et al. proposed a method utilizing modified adenoviruses, while Sakurai et al. developed a conditionally replicative adenovirus (OBP-1101) that integrates Ad35 fiber with the miR-142-3p regulatory system, resulting in a substantial reduction in false-positive rates (16, 17). However, OBP-1101 remains limited by interference from false-positive white blood cells during cervical cancer CTC detection, highlighting its shortcomings in practical applications (46). Furthermore, miR-142-3p expression is low in certain blood cell types, such as regulatory T cells, and may be upregulated in pancreatic ductal adenocarcinoma cells due to triptolide and quercetin, potentially restricting its broader applicability (47–49). Although telomerase activity is present in over 80% of tumor cells, approximately 20% of CTCs lack telomerase activity, thereby reducing the sensitivity of telomerase-based detection methods (50). Ji-Eun Hwang et al. improved infection efficiency using the Ad5/35E1aPSESE4 adenovirus in conjunction with PSA/PSMA transcription regulatory elements, but this approach is largely restricted to prostate cancer applications (51).

In contrast, our methodology employs the MUC1 promoter, providing a more targeted and specific approach for CTC detection. Initial experimental results demonstrate that the rAdF35-MUC1 achieves significantly enhanced infection efficiency and sensitivity in MUC1-positive breast cancer cells *in vitro*. Moreover, this viral vector effectively avoids nonspecific activation of the hTERT promoter. Clinical detection data further reveal that the average number of false-positive white blood cells generated by rAdF35-MUC1 is only 1.33, underscoring the method’s high practicality and potential for broad application in detecting various cancer types.

The limitations of this study encompass the small sample size of clinical specimens, thereby necessitating larger-scale clinical trials to substantiate the correlation between CTCs and breast cancer progression and prognosis. Additionally, future research should investigate the potential applications of the MUC1 promoter in other epithelial cancers, such as ovarian and lung cancers, to augment the clinical utility of CTC detection technologies in cancer screening. In conclusion, we have successfully engineered and validated rAdF35-MUC1, a novel adenovirus that detects CTCs in the blood of cancer patients with high sensitivity and specificity, offering a promising tool for early diagnosis and monitoring treatment efficacy.

## Data Availability

The original contributions presented in the study are included in the article/**Supplementary Material**. Further inquiries can be directed to the corresponding authors.
