# Supplementary material for: Enhanced detection of circulating tumor cells using a MUC1 promoter-driven recombinant adenovirus
Source: Front Oncol. 2025 Jan 16;14:1506968. doi: 10.3389/fonc.2024.1506968 (PMC11779711; doi:10.3389/fonc.2024.1506968)
Supplement: Supplementary file 1 [file DataSheet1.pdf]

Supplementary Material

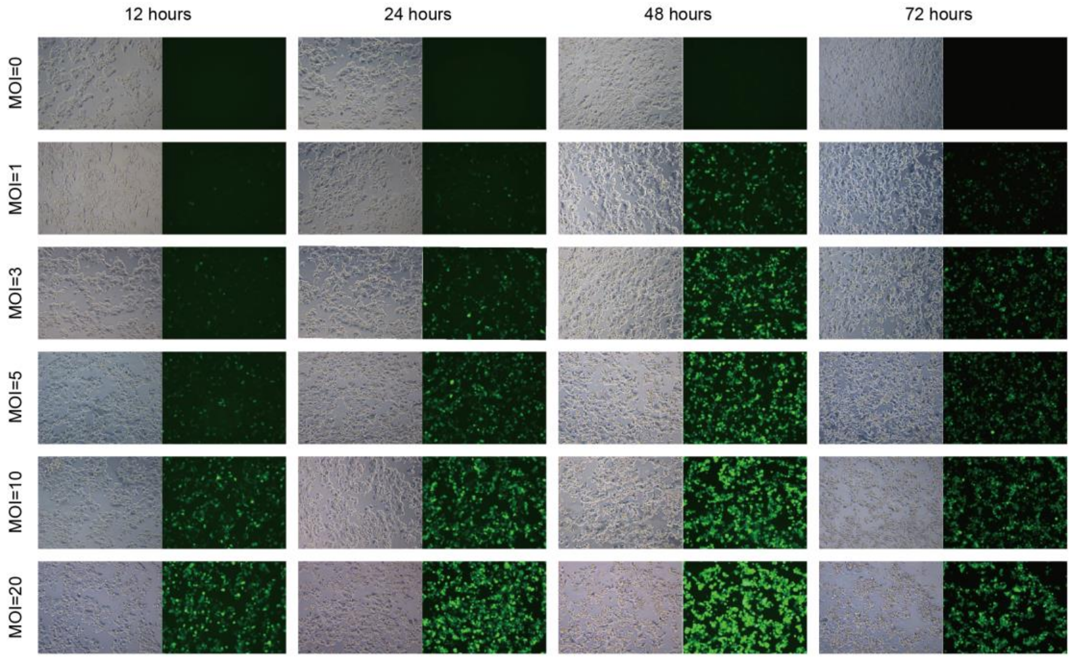

Supplementary Figure 1. MCF-7 cells were infected with rAdF35-MUC1 at designated multiplicities of infection (MOI = 0, 1, 3, 5, 10, 20) for various time points (12, 24, 48, and 72 h). Selected images taken at the indicated time points illustrate cell morphology by phase-contrast microscopy (left panels) and copGFP expression under fluorescence microscopy (right panels). Images were captured at 100X magnification.

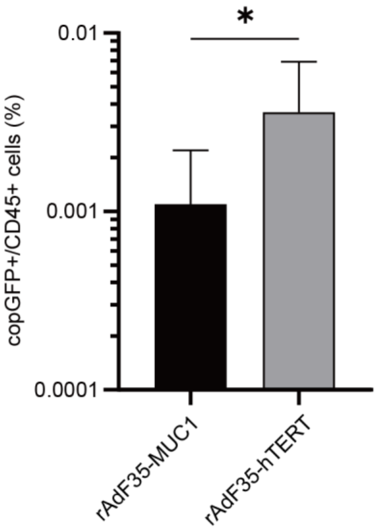

Supplementary Figure 2. Quantification of copGFP-positive PBMCs following infection with rAdF35-MUC1 and rAdF35-hTERT. The percentage of copGFP-positive PBMCs was plotted. Data are presented as mean ± SD; n = 10; $*p < 0.05$.

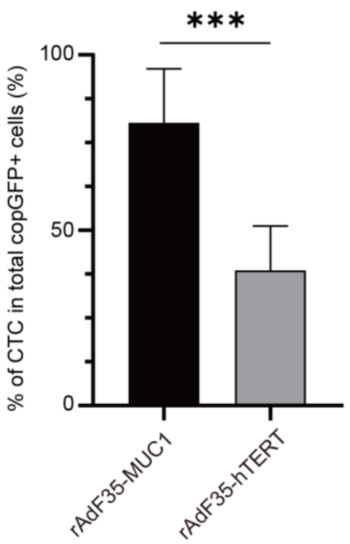

Supplementary Figure 3. Percentages of CTCs among total copGFP-positive cells in CTC-positive samples following infection with rAdF35-MUC1 and rAdF35-hTERT (10 samples for rAdF35-MUC1 and 7 samples for rAdF35-hTERT). The percentage of CTCs in total copGFP-positive cells was calculated by dividing the number of CTCs (copGFP+/CD45-) by the total number of copGFP-positive cells. The data are expressed as mean ± SD; n=15; ***$p < 0.001$.
